# Immunoclassification characterized by CD8 and PD-L1 expression is associated with the clinical outcome of gastric cancer patients

**DOI:** 10.18632/oncotarget.24037

**Published:** 2018-01-06

**Authors:** Weili Wang, Kuansong Wang, Zihua Chen, Ling Chen, Wei Guo, Ping Liao, Daniel Rotroff, Todd C. Knepper, Zhaoqian Liu, Wei Zhang, Howard L. Mcleod, Yijing He

**Affiliations:** ^1^ Department of Clinical Pharmacology, Xiangya Hospital, Central South University, Institute of Clinical Pharmacology, Central South University, Hunan Key Laboratory of Pharmacogenetics, Changsha, Hunan, China; ^2^ Department of Pathology, Xiangya Hospital, Central South University, Changsha, Hunan, China; ^3^ Department of General Surgery, Xiangya Hospital, Central South University, Changsha, Hunan, China; ^4^ Moffitt Cancer Center, DeBartolo Family Personalized Medicine Institute, Tampa, FL, USA; ^5^ Bioinformatics Research Center, North Carolina State University, Raleigh, NC, USA

**Keywords:** gastric cancer, immunoclassification, chemotherapy, Epstein-Barr virus (EBV)

## Abstract

**Background:**

Gastric cancer (GC) is a major cause of cancer deaths, especially in Eastern Asia. Current classification systems, including the WHO, Lauren, and TCGA, have clarified the pathological and molecular profiles of GC. However, these classifications lack an association with clinical outcome and guidance for medication selection.

**Objective:**

We aimed to identify a new immunoclassification for GC to better predict patient prognosis and aid in patient selection for immunotherapy.

**Results:**

For all samples, 35 were EBV positive (+) and 112 were EBV negative (-). EBV infection was associated with the number of CD3+ T cells (OR = 2.91 95% CI 1.27-6.68, *p* = 0.012) and PD-L1 expression in TME (OR = 2.57, 95% CI 1.13–5.82, *p* = 0.024). EBV+ patients showed a poor overall survival (OS) compared with EBV- patients (HR = 2.37; 95% CI, 1.03–5.41; *p* = 0.011). Importantly, WIR patients lived significantly shorter than SIR patients with high CD8+ T cells and low PD-L1 expression (HR = 3.37; 95% CI, 1.63–6.97; *p* = 0.015).

**Materials and Methods:**

147 formalin-fixed and paraffin-embedded (FFPE) samples of GC were obtained. Epstein-Barr virus (EBV) infection was measured. Immune markers including CD3, CD8 and PD-L1 were detected by immunohistochemistry (IHC) at tumor infiltration area (TI) and invasive margin area (IM) in tumor microenvironment (TME). PD-L1 expression was assessed by immunoreactive score (IRS) system. For immunoclassification, patients were classified into two subgroups: strong immunoreaction (SIR) and weak immunoreaction (WIR) defined by the number of CD8+ T cells and PD-L1 expression in TI.

**Conclusions:**

In this study, we suggest a new immunoclassification for gastric cancer which is associated with patient outcome and may provide a way to guide immunotherapy in the future.

## INTRODUCTION

Gastric cancer (GC) is the fifth most common cancer in the world and is a major cause of cancer death, especially in Eastern Asia [1]. According to the 2015 Cancer Statistics in China, GC ranked the second for both incidence and mortality rate [2, 3]. Although there are many choices for the treatment of GC patients, including surgery, chemotherapy, radiation therapy, and targeted therapy, the prognosis of GC patients is still unsatisfactory.[4–6] Inspiringly, rapid progress in the development of immune checkpoint inhibitors, such as those targeting CTLA-4, PD-1 and PD-L1, may offer new hope for the treatment of GC patients. However, the response rates of these drugs across cancers is quite low (about 20–40%), thus the identification of biomarkers to better select responders remains an important goal [7, 8, 9].

Current classification systems for GC include conventional clinicopathological classification (primarily WHO and Lauren classifications) and molecular classification (defined by The Cancer Genome Atlas (TCGA) in 2013) [10, 11]. However, the conventional clinicopathological classifications is intricate and has a high requirement for histopathological level. Besides only less than 10% of these classifications can predict patients’ prognosis and hardly can provide guidance for patients’ treatment. The association of molecular classifications with clinical outcomes is still undefined and under excavation now [12]. Moreover, with the development of immunotherapy, there is a clear need for a more refined GC classification that incorporates immune markers to aid in development of these therapies and ultimately improve survival from this deadly disease [11].

In this study, we assessed both activating and inhibitory immune factors in the TME, aiming to identify immune markers for a new immunoclassification (IMC) in GC which may better predict prognosis and provide guidance for therapy selection.

## RESULTS

### EBV expression correlated with PD-L1 upregulation in TME and survival

**Figure 1:**
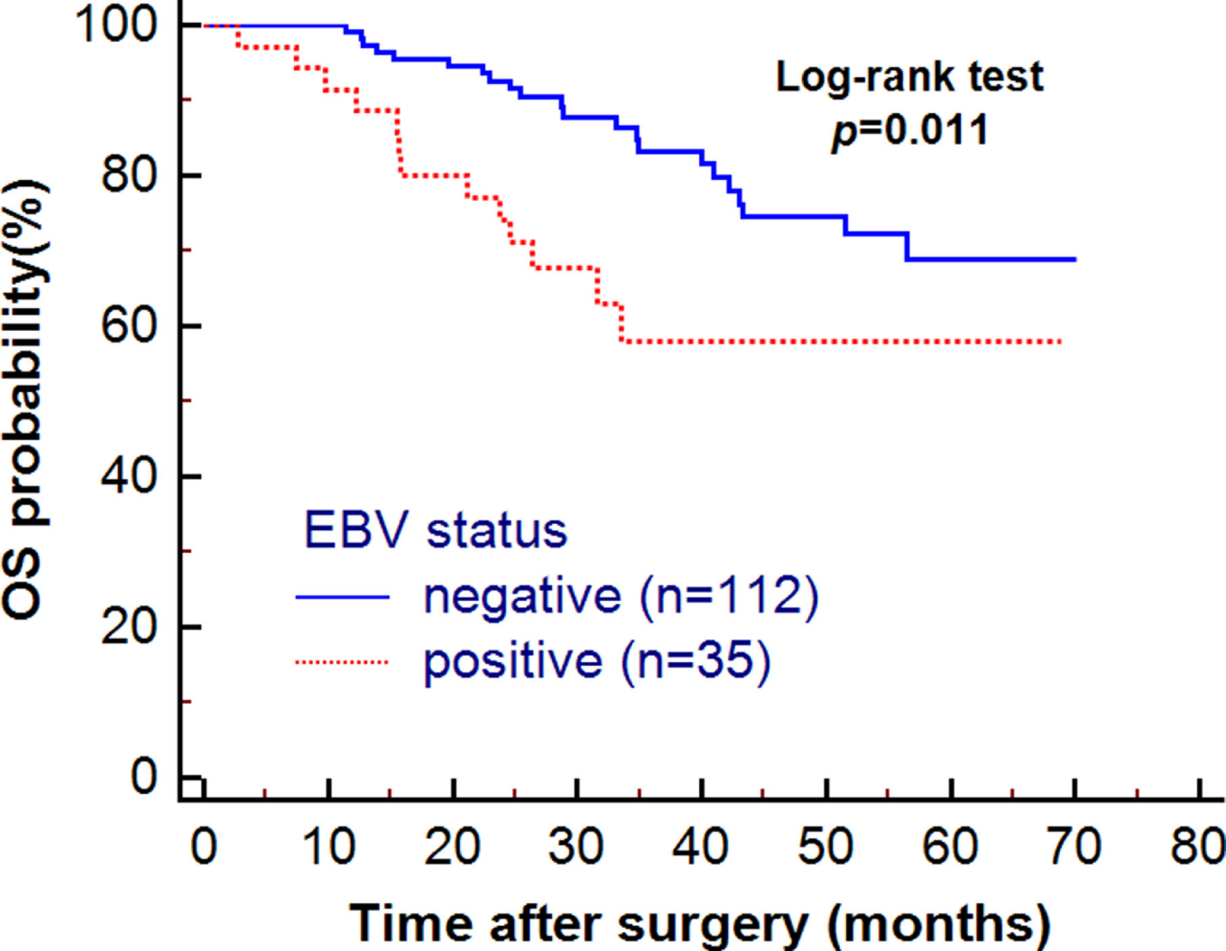
The association of patients’ overall survival (OS) with EBV infection

**Figure 2:**
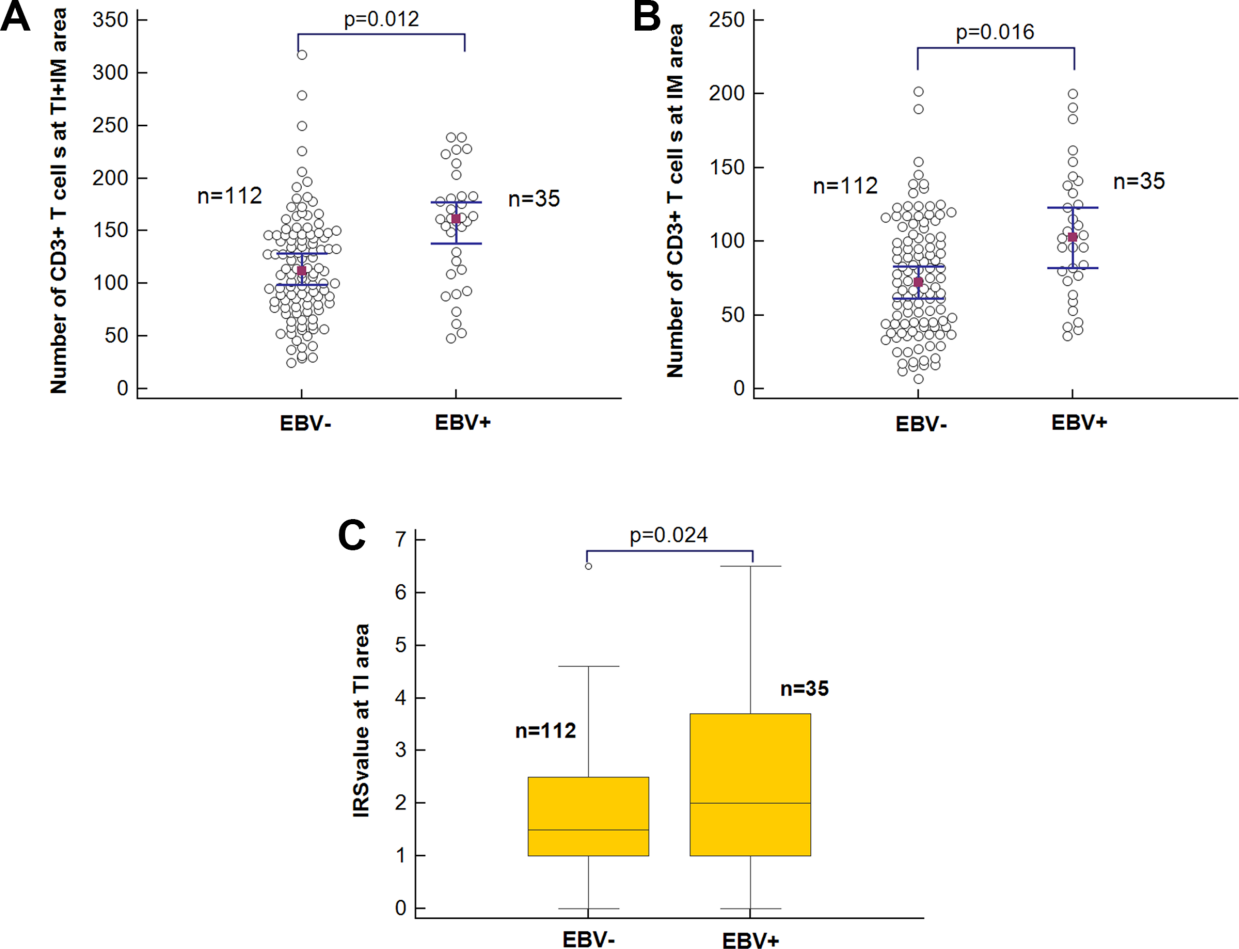
The association of EBV infection with immune status (**A**) CD3[TI+IM]; (**B**) CD3IM; (**C**) IRS value).

A total of 147 patients were enrolled in this retrospective study, including 35 EBV positive (+) and 112 EBV negative (-) patients. Kaplan–Meier analysis showed EBV- patients had a better overall survival (OS) compared with EBV+ patients (HR = 2.37; 95% CI, 1.03–5.41; *p* = 0.011) (Figure 1). EBV+ samples showed higher number of CD3+_[TI+IM]_ T cells (mean CD3+ T cells: 153[EBV+] vs 115 [EBV-]; OR = 2.91, 95% CI 1.27–6.68, *p* = 0.012) in TME (Figure 2A). Moreover, separate analysis showed EBV positivity tended to be associated with more CD3+ T cells at IM area (mean CD3+ T cells: 105 [EBV+] vs 75 [EBV-]; OR = 2.80, 95% CI 1.21–6.46, *p* = 0.016) (Figure 2B). However, no significant difference in the number of CD8+_[TI+IM]_ T cells was detected between EBV+ and EBV- patients (mean CD8+ T cell: 94[EBV+] vs 74[EBV-]; OR = 1.75, 95% CI 0.80–3.82, *p* = 0.16). Importantly, EBV infection was also significantly correlated with high level of PD-L1 expression at TI area (mean IRS value: 2.4 [EBV+] vs 1.7 [EBV-]; OR = 2.57, 95% CI 1.13–5.82, *p* = 0.024) (Figure 2C), which may be responsible to the prognosis distinction between EBV+ and EBV- patients.

### Immunoclassification of gastric cancer patients and clinicopathological characteristics in different groups

Five patients were eliminated for the lack of CD8 or PD-L1 expression data due to the failure of IHC. In total, 38 out of 142 (26.8%) patients were in SIR group, leaving the other 104 (70.7%) for WIR group (Table 1). There were no significant differences in age of onset, gender, drinking history, TNM stage, tumor location, EBV status, or the choice for chemotherapy between these two groups (*p* < 0.05). However, pylorus tumors (*n* = 99) showed a comparatively lower PD-L1 expression compared with tumors at other location (IRS-high group: 20.2% vs 39.1%, *r* = 0.18, χ^2^ *p* = 0.027).

**Table 1:** The clinical characteristics of GC patients within SIR and WIR subgroups

Clinical characteristics	Total,n (%) 142 ^a^ (100)	SIR,n (%) 38 (26.8)	WIR,n (%) 104 (73.2)	OR (95% CI)	Pearson χ2 p
median age	52	54	50		0.30
Gender Male Female	97 (68.3) 45 (31.7)	29 (76.3) 9 (23.7)	68 (65.4) 36 (34.6)	0.59 (0.25–1.37)	0.31
Drinking history Yes No	40 (28.2) 102 (71.8)	12 (31.6) 26 (68.4)	28 (26.9) 76 (73.1)	1.25 (0.56–2.82)	0.67
EBV status Positive negative	33 (23.2) 109 (76.8)	7 (18.4) 31 (81.6)	26 (25.0) 78 (75.0)	0.68 (0.27–1.72)	0.50
Tumor location					
Cardia/fundus	14 (9.9)	4 (10.8)	10 (9.6)		0.76
Gastric body	34 (24.1)	27 (27.0)	10 (23.1)		0.66
Pylorus	96 (68.1)	24 (64.9)	72 (69.2)		0.68
Multiple	3 (2.1)	1 (2.7)	2 (1.9)		
WHO grade Poor differentiation^b^ Middle-poor differentiation^b^ Middle differentiation^b^ High-middle differentiation^b^ Mucinous adenocarcinoma Signet-ring cell carcinoma	72 (50.7) 50 (35.2) 11 (7.7) 3 (2.1) 5 (3.5) 1 (0.7)	21 (55.3) 14 (36.8) 1 (2.6) 0 (0.0) 1 (2.6) 1 (2.6)	51 (49.0) 36 (34.6) 10 (9.6) 3 (2.9) 4 (3.8) 0 (0.0)		0.31
Invasion depth(T) T2 T3 T4a T4b	16 (11.3) 5 (3.5) 105 (73.9) 16 (11.3)	7 (18.4) 1 (2.6) 27 (71.1) 3 (7.9)	9 (8.7) 4 (3.8) 78 (75.0) 13 (12.5)		0.38
Lymph-node metastasis(N) N0 N1 N2 N3a N3b	24 (16.9) 43 (30.3) 36 (25.4) 32 (22.5) 7 (4.9)	8 (21.1) 12 (31.6) 11 (28.9) 7 (18.4) 0 (0.0)	16 (15.4) 31 (29.8) 25 (24.0) 25 (24.0) 7 (6.7)		0.44
TNM stage IIA IIB IIIA IIIB IIIC	10 (7.0) 23 (16.2) 34 (23.9) 38 (26.8) 37 (26.1)	2 (5.3) 11 (28.9) 9 (23.7) 9 (23.7) 7 (18.4)	8 (7.7) 12 (11.5) 25 (24.0) 29 (27.9) 30 (28.8)		0.15
Total TNM stage II III	33 (23.2) 109 (76.8)	13 (34.2) 25 (65.8)	20 (19.2) 84 (80.8)	0.46 (0.20–1.05)	0.07
Chemotherapy regimen^c^ FOLFOX XELOX DCF DOF/DOX ECF EOF/EOX	69 (48.6) 20 (14.1) 18 (12.7) 3 (2.1) 5 (3.5) 19 (13.4)	16 (42.1) 5 (13.2) 8 (21.1) 1 (2.6) 1 (2.6) 2 (5.3)	53 (51.0) 15 (14.4) 10 (9.6) 2 (1.9) 4 (3.8) 17 (16.3)		0.45 1.00 0.09 1.00 1.00 0.10

^a^: Five patients were excluded from the final analysis as the lack of IHC results of CD8 or PD-L1.

^b^: Tubular adenocarcinoma

^c^:FOLFOX: 5-FU+ leucovorin + oxaliplatin; XELOX: capecitabine + oxaliplatin; DCF: docetaxel + cis-platinum + 5-FU; DOF: docetaxel + oxaliplatin + 5-FU; DOX: docetaxel + oxaliplatin + capecitabine; ECF: epirubicin + cis-platinum + 5-FU; EOF: epirubicin + oxaliplatin + 5-FU; EOX: epirubicin + oxaliplatin + capecitabine.

### Immunoclassification associated with patients’ prognosis

**Figure 3:**
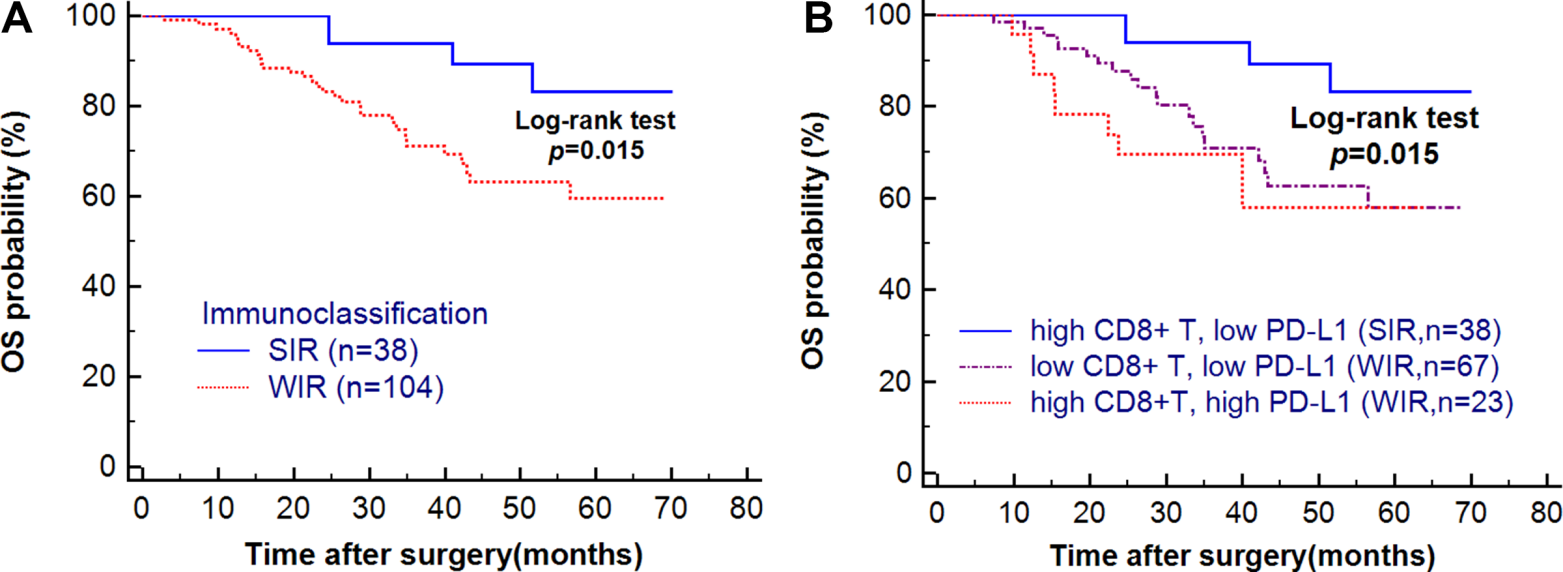
(**A)** Patients’ overall survival (OS) in different IMC subgroups. WIR patients lived shorter than SIR patients. (**B**) Patients’ OS in stratified IMC subgroups. High PD-L1 expression showed a bad effect in patients’ survival. (One subgroup didn’t show because of the small sample size).

Single-factor analysis for OS using Kaplan-Meier method showed that WIR patients lived significantly shorter than SIR patients (HR = 3.37; 95% CI, 1.63–6.97; *p* = 0.015) (Figure 3A). Stratified analysis showed that the subgroup with high CD8+ T cell and high PD-L1 expression in WIR patients had the worst prognosis (*p* = 0.015) (Figure 3B), implying that PD-L1 expression was significantly correlated with survival. Furthermore, cox regression analysis showed that male gender (HR = 3.88; 95% CI, 1.68–8.98; *p* = 0.002), stage III (HR = 8.56; 95% CI, 1.05–69.74; *p* = 0.046), EBV infection (HR = 2.36; 95% CI, 1.03–5.37; *p* = 0.044) and non-FOLFOX treatment regimens (HR = 2.94; 95% CI, 1.04–8.33; *p* = 0.043) were also associated with decreased OS (Supplementary Table 1).

### Immunoclassification had no effect on chemotherapy outcome

The most common treatment received was FOLFOX (47.6%). Among patients receiving FOLFOX, there was no difference in survival between SIR (HR = 0.32; 95% CI, 0.045–2.27; *p* = 0.29) (Supplementary Figure 1A) and WIR patients (HR = 0.82; 95% CI, 0.40–1.69; *p* = 0.59) (Supplementary Figure 1B). Similarly, there was no difference in survival between WIR and SIR patients who were treated with non-FOLFOX regimens.

## DISCUSSION

In this study, we provide evidence for a new IMC for gastric cancer based on the tumor-infiltrating CD8+ T cells and PD-L1 expression and demonstrate that WIR patients may experience an inferior OS compared with SIR patients.

Previous studies have showed that CD3+ and CD8+ T cells were associated with improved survival in gastric cancer [13], while PD-L1 expression was usually associated with poor patient prognosis [14, 15]. Co-localization of inflammatory response with PD-L1 expression in tumors supports an adaptive resistance mechanism of immune escape has been suggested to classify cancers [16–18]. It is also important to point out that a convenient classifier is needed for the best clinical use. In this study we jointly used the key component of tumor-infiltrating lymphocytes (TILs) which are CD8+ T cells (known as cytotoxic T cell, immune active factor) and PD-L1 expression (immune inhibitory factor) as the criteria to classify gastric cancer. SIR patients had a high number of CD8+ T cells and low PD-L1 expression indicating a status of strong immune activity. These patients may have a better capacity to fight against cancer cells. Conversely, the WIR patients had lower number of CD8+ T cells or null CD8+ T cells which characterized by high PD-L1 expression in tumor infiltrating area. Thus the IMC could be used to identify GC patients with different prognosis based on TME level. In the future, IMC may have utility for seeking responders of PD-1 blockades. Anti-PD-1 agents function through binding to PD-1 on tumor-reactive T cells and inhibiting the interaction between PD-1 and PD-L1, thereby stimulating the anti-tumor response of T cells [19]. In our study, high CD8+ T cell simultaneously accompanied by high PD-L1 expression out of WIR group may represent a subgroup best suited for PD-1/PD-L1 blockade. Because patients in this subgroup would have more drug targets for PD-1/PD-L1 to bind and more cytotoxic T cells to eliminate cancer cells, which were reflected by high PD-L1 expression and high CD8. Further, patients in this subgroup experienced the poorest survival in this study, underscoring a need for improved treatment options. Previous studies have demonstrated that PD-1 blockade could be effective in patients with low PD-L1 expression, and that this may be related to high CD8+ T cells. The IMC shown here may have future clinical application in predicting responses to immune checkpoint therapy, however clinical correlation and validation is needed.

This study also investigated the EBV subtype. The results showed that EBV+ patients experienced a shorter survival compared with EBV- patients. According to multiple studies, the relationship between EBV infection and patients’ prognosis is controversial. A meta-analysis of 4599 GC patients found that EBV+ patients have decreased mortality compared with EBV- patients after the adjustment of other factors [20]. However, there are other studies suggesting that the mortality risk of EBV+ patients is increased [21, 22]. Still, some studies show no prognostic role for EBV status. One study enrolled stage I-III GC patients and found that there was no significant difference of OS between the groups (*P* = 0.977) [23]. Mechanistically, studies found that EBV infection is correlated with increased infiltration of CD8+ T cells and mature dendritic cells [24–27]. These factors may partially contribute to antitumor immunity. While on the other side, EBV infection has been shown to be one of the intrinsic mechanisms for PD-L1 regulation and was associated with higher PD-L1 expression, including in our study. TCGA data also showed EBV positive GC displayed recurrent PIK3CA mutations, extreme DNA hyper methylation, and enhanced expression of JAK2, PD-L1, and PD-L2 [11]. Therefore, EBV infection in GC may trigger both positive and negative impact on cancer progression that may cause a complex influence on patient immune status and prognosis. This may partly explain the controversial role of EBV infection in GC. In our study, EBV induced immune inhibitory elements and was associated with inferior clinical outcome.

Using immune markers to classify cancers is an emerging field. In colorectal cancer, TILs, as measured by an immunoscore has become a method to predict patients’ prognosis and drug response. This score was measured by numeration of two lymphocyte populations (CD3/CD45RO, CD3/CD8 or CD8/CD45RO), in both the center of the tumor (CT) and in the invasive margin (IM) [28]. In melanoma, PD-L1 and TIL status were proposed to classify the TME into four different types, including Type I (TIL+, PD-L1+), Type II (TIL-, PD-L1-), Type III (TIL-, PD-L1+), Type IV (TIL+, PD-L1-).[16, 17] However, details about which cell type to be included in TIL and the threshold criterion are not defined yet.

Recently another immunoscore signature was reported specifically for GC.[29] A prognostic classifier named IS_GC_ for Chinese GC patients, which includes 5 immune features was developed using the LASSO Cox regression model. The 5-year DFS and OS were lower in the low-IS_GC_ group than those in the high-IS_GC_ group. In stage II and III GC patients, adjuvant chemotherapy significantly increased DFS and OS in the high-IS_GC_ group, but had no significant effect on the low-IS_GC_ group. Adjuvant chemotherapy demonstrated slight reduction the 2-year recurrence rate, mainly in the high IS_GC_ group. The predictive accuracy for prognosis and potential use for choosing patients who might benefit from adjuvant chemotherapy are the highlights of IS_GC_. However, limitation remain, that are not present in the IMC discussed here. First, the application for IS_GC_ in the clinic is remains a challenge due to the impracticality of identifying the expression of multiple markers in different areas (CT/IM) of such a heterogeneous cancer. Second, the association of IS_GC_ with PD-L1 expression is lacking, thus as an immunoscore it may have less utility in predicting response to immunotherapy, particularly as related to PD-1 blockade. Third, the association with TCGA molecular classification, particularly the EBV subtype is lacking.

In this study, we confirmed the impact of EBV infection on the expression of PD-L1 in TME and patients’ survival in GC. Furthermore, we provided a new immunoclassification of GC and assessed the influence of this classification on patients’ prognosis.

## MATERIALS AND METHODS

### Subjects

The Electronic Medical Records (EMR) of 454 GC patients from Hunan Cancer Hospital treated between 2009 and 2013 were assessed for eligibility. 147 patients with documented stage II or III GC with an onset age between 18 to 80 years old, without serious heart disease/ kidney disease/ metabolic disorders/ epilepsy/ brain metastases/ or immune deficiency were included. All patients had no known familial inherited cancer syndrome and received conventional chemotherapy after D2 gastrectomy. Patients’ demographic and treatment characteristics were collected from the EMR and overall survival time was collected by follow-up. Formalin-fixed and paraffin-embedded (FFPE) samples of these patients were obtained. The study was approved by ethics committee of the Central South University Institute of Clinical Pharmacology. Written informed consent for use of tumor samples for research was obtained from all patients prior to their surgery.

### Sample preparation

DNA extraction was implemented for detection of EBV infection [30, 31]. Tissue from 4 to 6 FFPE sections were transferred into 1.5 ml Eppendorf tubes. 1 ml of turpentine was added for deparaffinization and ophthalmic scissors were used to cut. Samples were spun for 20 seconds and centrifuged for 2 minutes at 13000 rpm. The upper liquid was carefully removed and the remaining tissue was washed by absolute ethanol once. After the complete evaporation of ethanol, DNA extraction was carried out according to the manufacturer’s protocol provided by Purelink™ Genomic DNA Kit (Invitrogen, USA). DNA concentration and purity were detected by BIOSPEC-NANO 230V analyzer. EP Tubes were encoded and stored at -20°C.

### EBV detection

PCR and gel electrophoresis were used to separate the EBV subgroups. Online software Primer 3.0 (http://frodo.wi.mit.edu) was used for primer design according to the EBNA-1 gene (a feature gene of EBV virus) sequence. The F primer was 5′-CCAGACAGCAGCCAATTGTC-3′ and the R primer was 5′-GGTAGAAGACCCCCTCTTAC-3′. DNA extracted from the B95–8 (EBV-positive Burkitt’s lymphoma) cell line was used as a positive control. Products of PCR were electrophoresed in a 2% agarose gel and then stained with ethidium bromide. If a 129 bp fragment was detected, it was classified into EBV+ group. The EBV+ results were subsequently validated by the Taqman real-time PCR and specific EBER-ISH method.

### Immunohistochemistry (IHC)

FFPE tissues were cut into 3–5 μm sections for hematoxylin and eosin (H&E) and IHC staining. For the IHC of CD3, CD8, and PD-L1, sections were deparaffinized in turpentine and rehydrated through a series of graded ethanol. CD3 and CD8 antigen retrieval was performed in a sodium citrate buffer (pH 6.0) in a microwave oven four times at 600 W for 8 min. PD-L1 antigen retrieval was performed in a diluted universal HIER antigen retrieval reagent(ab208572, Abcam, UK,1:10) using a pressure cooker for 3 minutes with full pressure (120°C). Endogenous peroxidase was inactivated by incubating the slides with 3% hydrogen peroxide. Nonspecific protein binding was blocked with normal goat serum (ZSGB-BIO, Beijing, China) for at least 1 hour. The sections were incubated with monoclonal antibody CD3 (ab-16669, Abcam, UK, 1:200), CD8 (ab-4055, Abcam, UK, 1:400), PD-L1 (ab-205921, Abcam, UK, 1:600), respectively, at 37°C (3 hours) or 4°C (overnight). Bound antibodies were detected by using a conventional streptavidin-biotin method according to manufacturer’s instructions (S-A/HRPkit, ZSGB-BIO, Beijing, China). The reaction was visualized by DAB+ Chromogen, and nuclei were counterstained using hematoxylin. Finally, slides were covered by coverslips using gelatin.

### Evaluation of immune status

**Figure 4:**
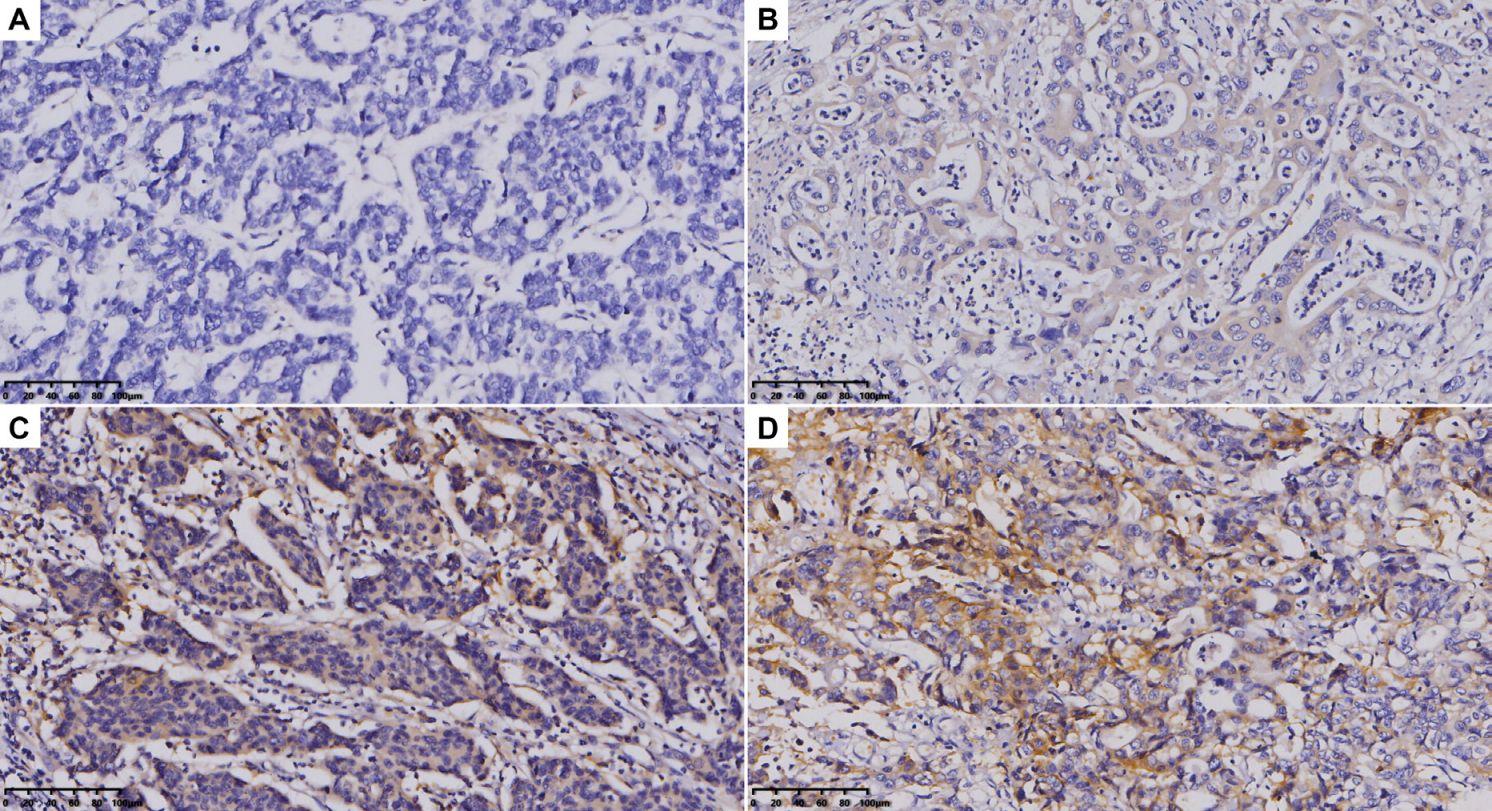
PD-L1 expression in gastric cancer tissues (**A**) 0+, no extracellular staining in the field which means no PD-L1 expression; (**B**) 1+, a slight brown extracellular staining in the field which means weak PD-L1 expression; (**C**) 2+, a brown extracellular staining in the field which means moderate PD-L1 expression; (**D**) 3+, an almost yellow extracellular staining in the field which means strong PD-L1 expression. Magnification: 400x. The antibody used for IHC of PD-L1 was Anti-PD-L1 antibody [28-8] ab205921, which corresponds to the extracellular domain of human PD-L1.

All IHC staining slides were scanned as panoramas by Automatic Digital Chip-scanner (KF-PRO-005, Kfbio, China). For the evaluation of PD-L1 expression, the percentage of positive cells were recorded with distinct extracellular staining intensity (0, no staining; 1+ weak/equivocal staining; 2+ moderate, definitive staining; 3+ strong, definitive staining) (Figure 4). Immunoreactive score (IRS) according to Remmele and Stegner [32] (Supplementary Table 2) was used to rate the expression level. For the evaluation of CD3 and CD8, 5 fields (radius = 150 μm) in the invasive margin area (IM) and 10 fields (radius = 150 μm) in the tumor infiltration area (TI) were selected (Supplementary Figure 2). Absolute numbers of positive cells were counted, averaged and classified as TI or IM. All slides were examined and scored independently by two investigators.

### Immunoclassification for gastric cancer

In this study, we established the IMC of GC based on the number of cytotoxic T cells (CD8+ T cells) and level of PD-L1 expression at TI area. Median amount of CD8+ T cells (24) at TI was used to classify high/low cytotoxic T cell infiltration. As for the evaluation of PD-L1 expression, the cutoff value of IRS to define IRS-high (high PD-L1 expression) and IRS-low (low PD-L1 expression) was 3.[32] For IMC, two groups were identified. Patients with high CD8+ infiltration and low PD-L1 expression were classified into Strong Immune Reaction (SIR) group, while the left were classified into Weak Immune Reaction (WIR) group (Table 2).

**Table 2:** The immunoclassification of gastric cancer

Immunoclassification	CD8 + T cell	PD-L1 expression
SIR	high	low
	high	high
WIR	low	low
	low	high

### Statistical analysis

Statistical analysis was performed using SPSS version 13.0 and MedCalc^®^ version 11.4.2.0. The significance of clinic characteristics and IMC among groups was tested by the χ2 test and Fisher’s exact test. Kaplan–Meier method was used for single-factor survival analysis among different groups and log-rank tests were carried out to compare the differences between survival curves. Maximum likelihood estimates of hazard ratios (HRs), 95% confidence intervals (CIs), and likelihood ratio statistics in Cox proportional hazards models were obtained with a limited backward-selection procedure used to adjust for potential confounding covariates. Statistical significance was accepted when *P* < 0.05. All the *P* values presented were two-sided.

## SUPPLEMENTARY MATERIALS FIGURES AND TABLES


