# Supplementary material for: Immunoclassification characterized by CD8 and PD-L1 expression is associated with the clinical outcome of gastric cancer patients
Source: Oncotarget. 2018 Jan 6;9(15):12164–73. doi: 10.18632/oncotarget.24037 (PMC5844736; doi:10.18632/oncotarget.24037)
Supplement: Supplementary file 1 [file oncotarget-09-12164-s001.pdf]

Immunoclassification characterized by CD8 and PD-L1 expression is associated with the clinical outcome of gastric cancer patients

SUPPLEMENTARY MATERIALS

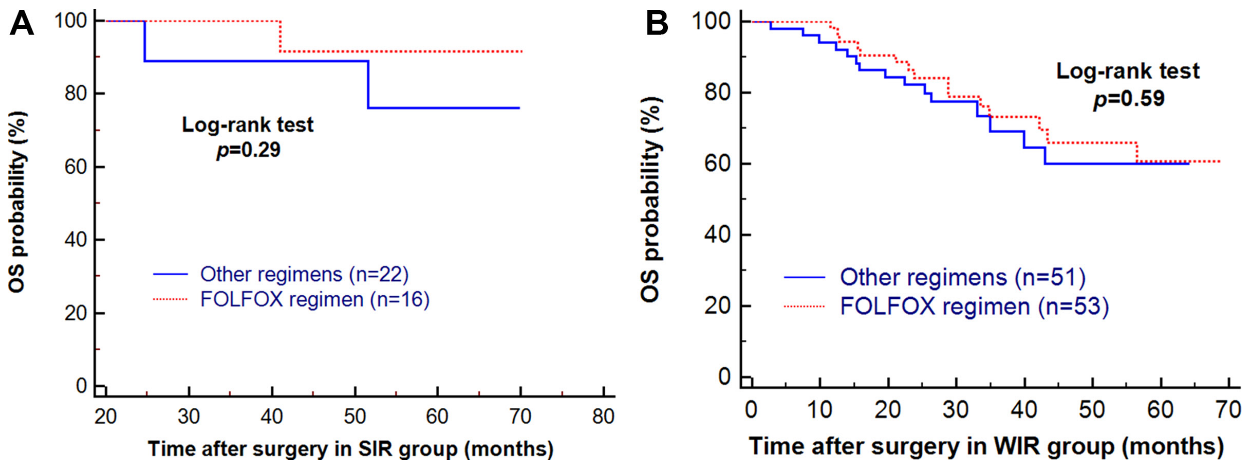

Supplementary Figure 1: The effect of FOLFOX regimen on OS within different IMC groups. (a. SIR; b. WIR).

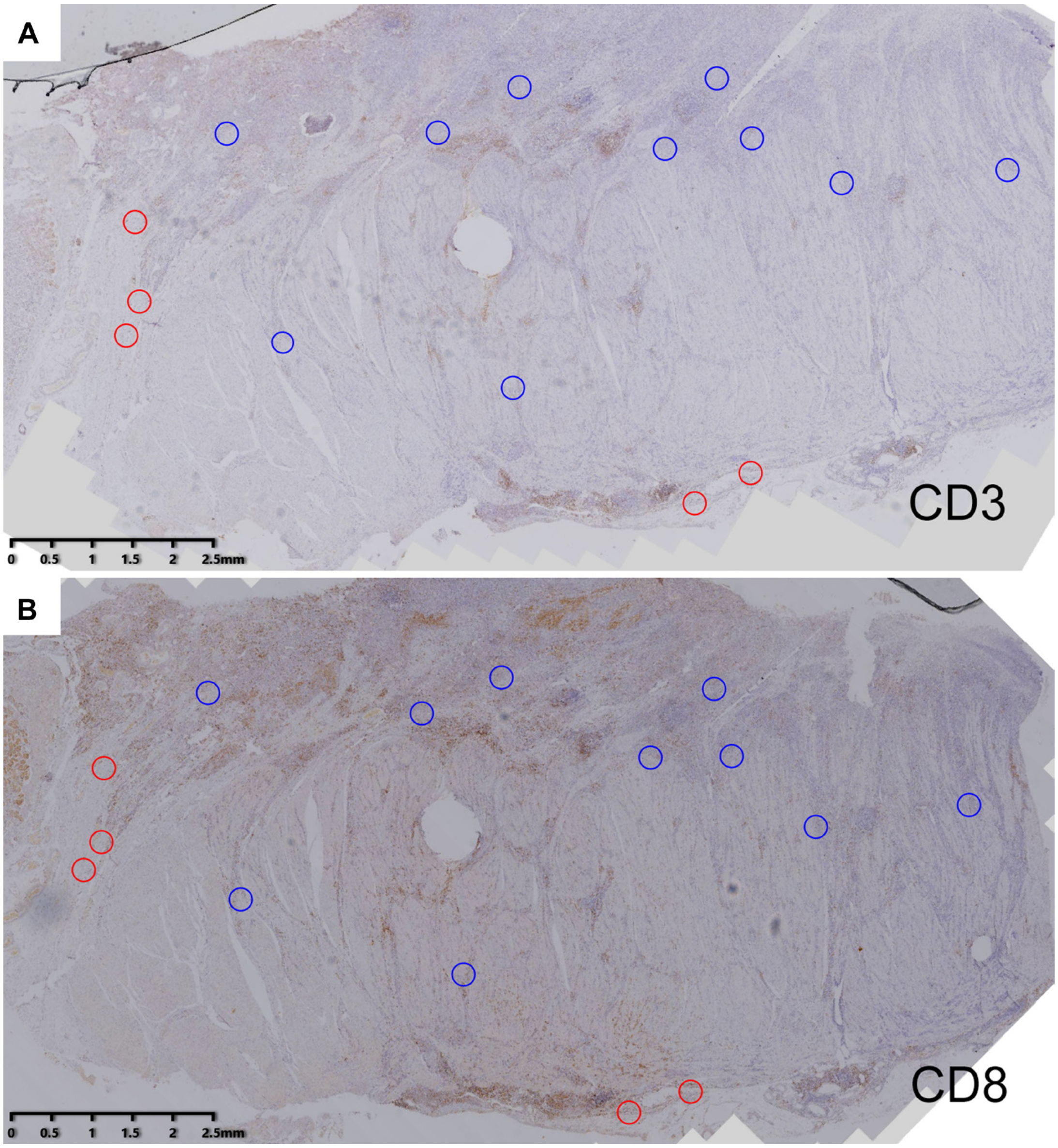

Supplementary Figure 2: Evaluation of positive CD3+ and CD8+ T cells at tumor infiltration (TI) area or invasive margin (IM) area. The blue circle stands for TI and red circle stands for the IM. Same areas were chosen in CD3 and CD8 marked IHC sections.

Supplementary Table 1: The cox proportional-hazards regression analysis for patients' survival

Covariate	P	HR	95% CI of HR
Onset age > 60	0.173	1.91	0.76 to 4.82
gender = male	0.002	3.88	1.68 to 8.98
IMC = SIR	0.018	0.22	0.06 to 0.77
EBV = positive	0.044	2.36	1.03 to 5.37
TNM stage = III	0.046	8.56	1.05 to 69.74
WHO grade = middle-poor differentiation	0.025	0.31	0.11 to 0.86
WHO grade = middle differentiation	0.319	0.44	0.09 to 2.18
WHO grade = high-middle differentiation	0.973	0.00	0.00 to 454E +267
WHO grade =signet-ring cell carcinoma	0.986	0.00	0.00 to 10.1E + 303
WHO grade = mucinous adenocarcinoma	0.466	0.42	0.04 to 4.23
Tumor location = cardia/fundus	0.492	0.46	0.050 to 4.21
Tumor location = gastric body	0.421	0.68	0.26 to 1.74
Tumor location = multiple	0.230	2.72	0.54 to 13.76
FOLFOX = yes	0.043	0.34	0.12 to 0.96
XELOX = yes	0.778	0.82	0.21 to 3.18
DCF DOF DOX = yes	0.660	1.26	0.45 to 3.55
ECF EOF EOX = yes	0.870	0.90	0.27 to 3.05

Supplementary Table 2: Immunoreactive score (IRS) according to Remmele and Stegner

Staining intensity		Percentage of positive cells		Valuea	Classification
0	**X**	0 (negative)	=	0-1	0
1		1 (<10% positive cells)		2-3	1
2		2 (10%-50% positive cells)		4-8	2
3		3 (51%-80% positive cells)		9-12	3
		4 (> 80% positive cells)			

[a]A cutoff value of 3 was used to define IRS-high (high PD-L1 expression) or IRS-low (low PD-L1 expression)
